# On the evaluation of hydrogen evolution reaction performance of metal-nitrogen-doped carbon electrocatalysts using machine learning technique

**DOI:** 10.1038/s41598-021-00031-0

**Published:** 2021-11-09

**Authors:** Alireza Baghban, Sajjad Habibzadeh, Farzin Zokaee Ashtiani

**Affiliations:** 1 Chemical Engineering Department, Amirkabir University of Technology (Tehran Polytechnic), Mahshahr Campus, Mahshahr, Iran; 2 Surface Reaction and Advanced Energy Materials Laboratory, Chemical Engineering Department, Amirkabir University of Technology (Tehran Polytechnic), Tehran, Iran; 3 Chemical Engineering Department, Amirkabir University of Technology (Tehran Polytechnic), Tehran, Iran

**Keywords:** Energy science and technology, Engineering, Materials science, Nanoscience and technology

## Abstract

Single-atom catalysts (SACs) introduce as a promising category of electrocatalysts, especially in the water-splitting process. Recent studies have exhibited that nitrogen-doped carbon-based SACs can act as a great HER electrocatalyst. In this regard, Adaptive Neuro-Fuzzy Inference optimized by Gray Wolf Optimization (GWO) method was used to predict hydrogen adsorption energy (ΔG) obtained from density functional theory (DFT) for single transition-metal atoms including Sc, Ti, V, Cr, Mn, Fe, Co, Ni, Cu, Zn, Zr, Nb, Mo, Tc, Ru, Rh, Pd, Ag, Cd, Hf, Ta, W, Re, Os, Ir, Pt, and Au embedded in N-doped carbon of different sizes. Various descriptors such as the covalent radius, Zunger radius of the atomic d-orbital, the formation energy of the single-atom site, ionization energy, electronegativity, the d-band center from − 6 to 6 eV, number of valence electrons, Bader charge, number of occupied d states from 0 to − 2 eV, and number of unoccupied d states from 0 to 2 eV were chosen as input parameters based on sensitivity analysis. The R-squared and MSE of the developed model were 0.967 and 0.029, respectively, confirming its great accuracy in determining hydrogen adsorption energy of metal/NC electrocatalysts.

## Introduction

Hydrogen has been treated as a renewable energy resource, diminishes fossil resource consumption, and coping with global warming^1,2^. Water splitting is a competent technique to produce hydrogen^3^. It exploits renewable resources to provide electricity^4,5^. This process is basically performed in acidic and alkaline solutions. The reported reaction rate of the HER in acidic solutions is approximately 2–3 orders of magnitude higher than that in alkaline solutions^6,7^. Researchers and experimentalists have primarily investigated acidic HER in terms of theoretical and experiments, but the energetics and kinetics aspects of alkaline HER, especially in computational chemistry, have been neglected^8^. Hence developing design principles for alkaline HER electrocatalysts has been a challenging subject in recent years^8^.

However, to employ water splitting, it is required to tackle a number of challenges, such as finding an abundant, affordable, and stable catalyst for use in place of effective hydrogen evolution reaction (HER) catalysts, such as precious metals (e.g., Pt)^8–11^. Metal compounds, including sulfides, phosphides, and nitrides, have recently been found to be promising in HER^12–18^. Moreover, research has shown the viable catalytic performance of two-dimensional substances, including graphene-supported metals^19–22^ and the dichalcogenides of transition metals^23–27^. Graphene-supported single-atom catalyst (SAC) transition metals enjoy significant advantages, including full metal utilization, large chemical property turnability, and significant activity. Graphene serves as a substrate of high conductivity and stability and provides a great surface area for the support of single atoms^28^.

So far, remarkable studies have been carried out in designing advanced non-noble metal catalysts to substitute noble metal-based catalysts such as Pt or Ru-based catalysts^29–31^. These researches introduced some structures with heteroatoms (i.e., N, P, S, or B) doped carbon materials as one of the most promising substitutes for the HER process, owning their remarkable features such as low cost, high activity, and robust stability.

For example, it is possible to dope nitrogen to tune graphene further and achieve enhanced performance. In general, single atoms appear in the vacancies of N-doped or pristine graphene support. As many as three or four M–N or M–C covalent bonds may stabilize such structures^32,33^. A large number of studies investigated and characterized single atoms in different areas (e.g., HER); however, their catalytic performance and design factors are yet to be adequately clarified. It is believed that SAC performance is dependent on the transfer of charge between the metals of atomic dispersion and substrates and the chemical bonding^34–36^.

Researchers have applied several techniques, such as SAC element change, SAC coordination environment alternation, and the S, P, and N doping of the substrate, so that SAC catalytic performance could be tuned^37^. Apart from doping graphene with non-metals, changing the size of the substrate (from a two-dimensional periodic structure into graphene nano-molecules with different sizes) can improve the tuning controllability of the electronic structure of the substrate. Furthermore, research has shown that macrocycle single atoms have high stability and may exhibit high molecular catalytic activity^38,39^. The use of the electronic structure of the substrate to modular SAC properties is an efficient method. Thus, the present study adopted it to evaluate SACs in terms of HER performance by transforming a substrate of metallic graphene into a nano- or macro-cyclic substrate with molecule-like states.

The quantum chemistry calculation is a powerful technique to investigate theoretically chemical phenomena like reaction, adsorption/desorption, electrical and thermodynamic properties of chemical structures^40–43^. Moreover, machine learning approaches have recently been applied in different areas such as chemistry, catalyst, energy, chemical processes, etc.^44–49^. Support vector machine (SVM)^50^, artificial neural network (ANN)^51,52^, fuzzy logic system (FLS)^53^, and adaptive neuro-fuzzy inference system (ANFIS)^54,55^ are the most familiar categories of machine learning which can be optimized by different optimization algorithms such as particle swarm optimization (PSO)^48^, genetic algorithm (GA)^56^, gray wolf optimization (GWO)^46^, imperialist competitive algorithm (ICA)^57^, teaching learning-based optimization (TLBO)^58^, etc. In the present contribution, we predict hydrogen adsorption energy (ΔG) obtained from density functional theory (DFT) for single transition-metal atoms embedded in N-doped nanographene of different sizes using optimized GWO-ANFIS approach.

## Computational methods

### Density functional theory (DFT)

The present study employed the Vienna *ab inito* simulation package (VASP) to carry out the spin-polarized calculations of density functional theory (DFT). The Perdew–Burke–Ernzerhof (PBE) generalized gradient approximation (GGA) functional was exploited to represent the electron exchange–correlation^59^. Also, the projector-augmented wave (PAW) technique was used to describe the interaction of ions with electrons. The present work set the plane-wave cutoff to 400 eV, performing geometry relaxation using a conjugate gradient technique until the interatomic forces fell below 0.025 eV/A. Moreover, the Grimme’s semi-empirical dispersion-corrected density functional theory (DFT-D2) was applied to consider weak interactions with great accuracy.

Graphene supper cells of 5 × 6 sizes with fifty-four C atoms were used to model N-doped graphene-supported SACs. A 20-A vacuum was applied to the two-dimensional monolayer of N-doped graphene in the z-direction. It was periodic on the XY plane. Also, a (3 × 3 × 1) Monkhorst–Pack *k*-point mesh was used to sample the Brillouin zone.

**Figure 1:**
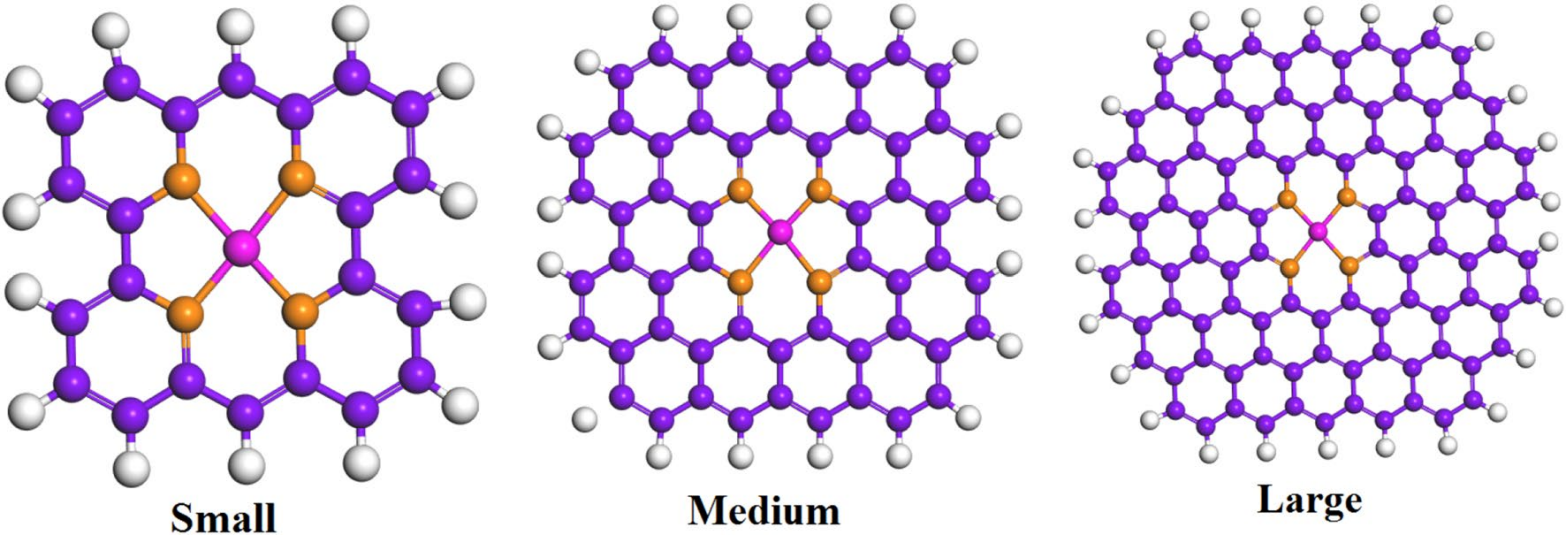
Structures of metal/N-doped carbon of different sizes: Key: C, purple; H, white; N, orange; metal, pink.

The modeling of N-doped nanographene-supported SACs (various nanographene sizes) was carried out within 30 × 30 × 30 A cubes, where a SAC was situated at the center of a nanographene. As shown in Fig. 1, the numbers of C atoms in the small, medium, and large nanographene were 22, 56, and 102, respectively. Also, the *k* space was sampled using the Γ point.

The present study obtained the adsorption energy of hydrogen as ${\Delta \mathrm{E}}_{H}=\mathrm{E}\left(\mathrm{catalyst}+\mathrm{H}\right)-\mathrm{E}\left(\mathrm{catalyst}\right)-\frac{1}{2}\mathrm{E}({\mathrm{H}}_{2})$, in which the term $\mathrm{E}\left(\mathrm{catalyst}+\mathrm{H}\right)$ denotes the total of the catalyst energy and the energy of a H atom that is adsorbed, the term $\mathrm{E}\left(\mathrm{catalyst}\right)$ stands for the total catalyst energy, and the term $\mathrm{E}({\mathrm{H}}_{2})$ is the total gaseous H_2_ molecule energy. Moreover, for the adsorption of H, the Gibbs free energy was calculated as $\Delta {\mathrm{G}}_{H}=\Delta {\mathrm{E}}_{H}+\Delta {\mathrm{E}}_{ZPE}-\mathrm{T}\Delta {\mathrm{S}}_{H}$, in which $\Delta {\mathrm{E}}_{ZPE}$ denotes the zero-point energy difference of the adsorbed H atom and the H atom of the gaseous H_2_ molecule, while $\Delta {\mathrm{S}}_{H}$ stands for the difference in entropy between the adsorbed H and gaseous ½H_2_ in standard conditions. Furthermore, the vibrational frequencies were summed up over normal models to obtain the zero-point energy as ${\mathrm{E}}_{ZPE}=\frac{1}{2}\sum h\omega$.

The NIST database was utilized to obtain the free H_2_ molecule entropy at 1 atm and 298.15 K^60^. The present work derived the SAC formation energy as ${\mathrm{E}}_{f}=\mathrm{E}\left(\mathrm{catalyst}\right)-\mathrm{E}\left(\mathrm{substrate}\right)-\mathrm{E}(\mathrm{TM})$, in which the term $\mathrm{E}\left(\mathrm{substrate}\right)$ denotes the total N-doped graphene energy, while $\mathrm{E}\left(\mathrm{TM}\right)$ stands for the total transition metal atom energy. The formation is desirable when the formation energy is negative.

### Adaptive neuro-fuzzy inference system (ANFIS)

The adaptive neuro-fuzzy inference system (ANFIS) was developed by Jang^61^. It integrates the capabilities of the ANN, and FIS approaches to cope with the disadvantages of individual ANN and FIS, e.g., membership function definition sensitivity and overfitting. The Sugeno FIS is the most commonly used technique in ANFIS training. It determines the model parameters by using a robust learning framework^62^. In general, the structure of ANFIS involves five layers. The first layer applies the generalized Gaussian membership function *μ* to the inputs in order to generate the output as:

$${Out}_{1i}=\mu {A}_{i}\left(x\right), i=1, 2$$

1 $${Out}_{1i}=\mu {B}_{i}\left(x\right), i=3, 4.$$

In which ${A}_{i}$ and ${B}_{i}$ are the membership values, while

2 $$\mu \left(x\right)={e}^{-({x-\frac{{p}_{i}}{{\sigma }_{i}})}^{2}},$$

where ${p}_{i}$ and ${\sigma }_{i}$ represent the sets of hypothesis parameters. Then, the node output in the second layer is obtained as:

3 $${Out}_{2i}=\mu {A}_{i}\left(x\right)\times \mu {B}_{i-2}\left(y\right).$$

Subsequently, the third layer normalizes the output of the previous layer as:

4 $${Out}_{3i}={\overline{W} }_{i}=\frac{{\omega }_{i}}{\sum_{i=1}^{2}{\omega }_{i}}.$$

The fourth layer subjects the output of the previous layer to adaptive nodes:

5 $${Out}_{4i}={\overline{w} }_{i}{f}_{i}={\overline{w} }_{i}({p}_{i}x+{q}_{i}y+{r}_{i})$$

in which *p, q,* and *r* stand for the consequent parameters of node *i*. Eventually, the model output is obtained as:

6 $${Out}_{5i}=\sum_{i}{\overline{w} }_{i}{f}_{i}.$$

The membership function parameters should be optimally determined during the training process. There are various optimization algorithms such as genetic algorithm (GA), particle swarm optimization (PSO), imperialist competitive algorithm (ICA), gray wolf optimization (GWO), etc., which can be coupled with ANFIS to find the best tuning parameters. Consequently, for complex problems, especially in quantum chemistry and molecular modeling, this approach can help chemists to have a simple-to-apply model by using the combination of learning, adaptability and nonlinear problem-solving features of artificial neural networks plus the significant notions of approximate reasoning and treatment of information suggested by the fuzzy set theory.

### Gray wolf optimization

Mirjalili et al. developed the gray wolf optimization (GWO) algorithm with a hierarchical architecture based on the social hunting behavior of wolves^63^. GWO has a population-based framework and identifies the optimal solution straightforwardly. It incorporates four groups of wolves, including alpha, beta, delta, and omega wolves, for hierarchical leadership simulation. Prey search, besiege, and hunt are the primary hunting steps. Alpha wolves can be either male or female and serve as the leaders. They manage the herd in, for example, resting and hunting. The beta wolves provide help to the alpha wolves with decision-making and may be promoted to the alpha group. The delta group involves baby-care, hunter, and older wolves. The omega wolves have the lowest rank of the hierarchical structure. They do not contribute to decision-making.

Alpha, beta, and delta wolves are employed to perform optimization. An alpha wolf is selected to lead the algorithm, and a beta wolf and a delta wolf are selected to contribute to the leadership. They are followed by the remaining wolves of the herd. The prey position and wolf position are mathematically formulated as:

7 $$\overrightarrow{D}=|\overrightarrow{C}.{\overrightarrow{X}}_{p}(t)-\overrightarrow{X}t|$$

and

8 $$\overrightarrow{X}\left(t+1\right)={\overrightarrow{X}}_{p}\left(t\right)-\overrightarrow{A}.\overrightarrow{B},$$

Respectively, where $\overrightarrow{A}$ and $\overrightarrow{B}$ denote the coefficient vectors, $\overrightarrow{X}$ is the wolf position vector, and ${\overrightarrow{X}}_{p}$ denotes the prey position vector. Also, *t* represents the iteration number. Vectors A and C are found as:

9 $$\overrightarrow{A}=2\overrightarrow{a}.{\overrightarrow{r}}_{1}-\overrightarrow{a},$$

10 $$\overrightarrow{C}=2{\overrightarrow{r}}_{2}.$$

In which the *a*-components undergo a linear reduction from 2 to 0 in iterations and the random vectors ${\overrightarrow{r}}_{1}$ and ${\overrightarrow{r}}_{2}$ vary from 0 to 1. The position of the prey is approximated by the alpha, beta, and delta wolves, with the remaining wolves randomly updating their positions around the prey. The wolves besiege the prey, and the alpha wolf makes an attack. The solutions are evaluated in suitability, selecting the top three solutions are the alpha, beta, and delta wolves, respectively. This process continues to be iterated, updating the positions of the wolves until the discontinuance criterion has been met. The final alpha wolf position is selected as the optimal solution.

## Results and discussion

### Screening N-doped graphene-supported TM as HER SACS

**Figure 2:**
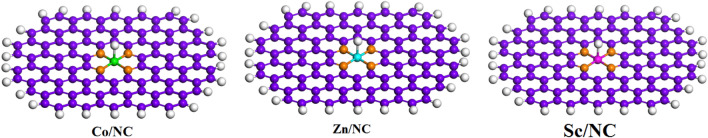
Optimized structures of H adsorption on the metal/NC: C, purple; H, white; N, orange; Co, green; Sc, pink; Zn, blue.

The first HER step is the Volmer step that results in the adsorption of H^64,65^. The Gibbs free energy in the adsorption of hydrogen is a good HER descriptive factor in a large number of catalysts^66^. Therefore, the adsorption of H was evaluated at N-doped graphene-supported 3d, 4d, and 5d single TM atoms. Figure 2 shows the optimized adsorption geometries of H at single graphene-supported TM atoms. It was observed that H was properly adsorbed onto the top of TM atoms. In addition, it was discovered that TM atoms had in-plane positions on graphene before and after hydrogen adsorption for most SACs (e.g., Co-NG). Moreover, both early and late transition metals, in particular the elements of groups 3 and 12, had out-of-plane positions (e.g., Sc-NG and Zn-NG). An explanation is the greater early TM radius and lower nitrogen-metal interaction for NG and the late TM.

**Figure 3:**
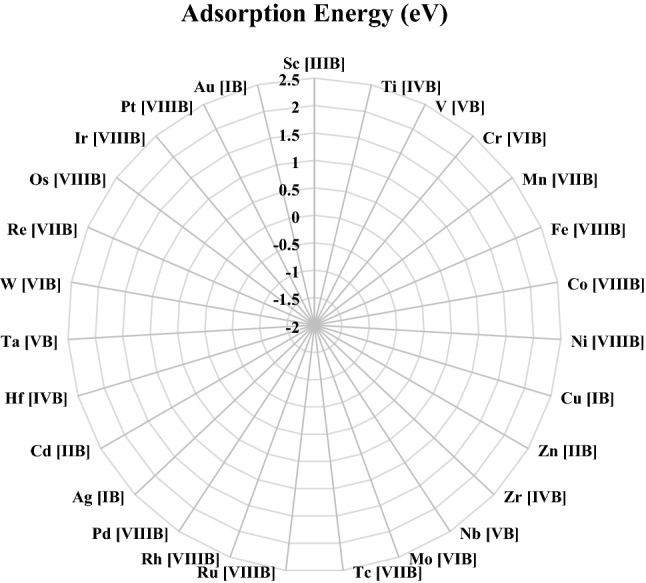
Comparison of hydrogen adsorption energy different metal/NC structures.

Figure 3 depicts the Gibbs free energy results for the adsorption of H. As can be seen, the largest negative adsorption energy occurs on the early transition metals. It becomes positive (weaker) from the left side to the right side. The H adsorption energy of group 10 is dramatically higher than that of group 9, leading to significantly undesirable adsorption onto the transition metal atoms of groups 10 and 11. Also, H adsorption increases for atoms in a lower position in each group (Mo > Cr, for example). This is specifically the case with groups 3–9. Research has reported the same trend for other SAC systems^67^.

The calculated free energy results agree with earlier SAC works^36,37^, and the present study sampled a larger number of elements. It should be noted that the free energy values could be dependent on the functional (Y@N_4_ SAC, for example)^36^. The present study, however, focuses on overall trends. N-doped graphene-supported Co has been known as the most efficient catalyst in HER based on the Gibbs free energy. Several works experimentally demonstrated that it had significant HER performance^35,68^. As a result, the proposed computational technique is verified. Furthermore, Ir, Rh, Fe, V, and SC are assumed to have good catalytic activity in NG as their Gibbs's free energy values are almost zero. Cd was not considered an efficient catalyst as it has a dramatically smaller negative SAC formation energy and thus lower stability than others.

### HER activity turning through graphene size

HER process in acid can be performed in three steps, as shown below.

11 $${{H}_{3}O}^{+}+e \to {H}^{*}+{H}_{2}O \left(Volmer \,reaction\right),$$

12 $${2H}^{+}+e \leftrightarrow {H}_{2}^{*} \left(Tofel\, reaction\right),$$

13 $${{H}_{3}O}^{+}+e+{H}^{*}\to {H}_{2}^{*}+{H}_{2}O \left(Heyrovsky\, reaction\right),$$

where H^*^ and ${H}_{2}^{*}$ stand for the hydrogen atom and molecule adsorbed onto a surface atom, respectively. As seen in Eqs. (11) to (12), one hydrogen from the hydronium molecule is adsorbed on the catalyst surface, and radical hydrogen is formed. From the Tafel reaction, a hydrogen molecule can be created from the reaction of two radical hydrogens.

The rate‐determining step (RDS) in HER process is the adsorption of hydrogen in Volmer–Heyrovsky pathway, and the relative importance of the energy barrier in the Volmer reaction is much higher than the Heyrovsky reaction.

**Figure 4:**
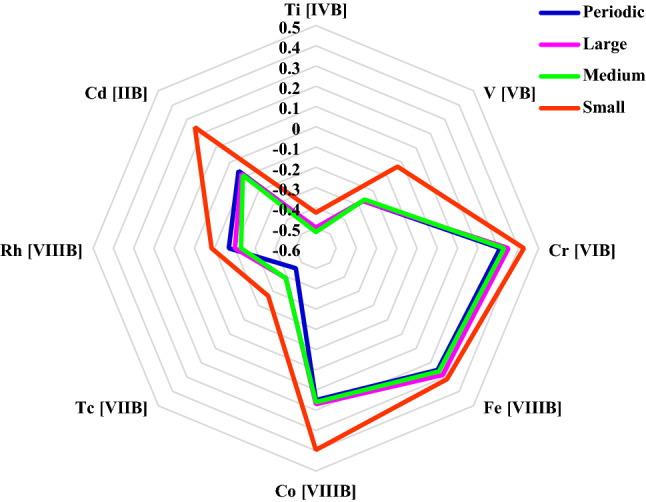
Comparison of hydrogen adsorption energy for different sizes of metal/NC structure.

According to Fig. 3, the Gibbs free energy values of elements can have significant differences (by more than 3 eV for TM SAC on NG). The present study systematically explored the effects of the graphene size on the Gibbs free energy to be further turned toward zero. Three hydrogen-terminated N-doped nanographene structures with successively smaller sizes were employed, including small twenty-two C atoms), medium (fifty-six C atoms), and large (102 C atoms), as shown in Fig. 1. The SACs supported by N-doped nanographene of the three sizes along with those in the extended N-doped graphene, are illustrated in Fig. 4. It should be noted that solely the SACs with Gibbs free energy values of − 0.5 to 0.5 eV are illustrated. The nanographene of large and medium sizes showed no significant changes, while small nanographene exhibited a largely weakened H binding (by 0.1–0.3 eV). As mentioned, the exchange current density *j*_*0*_ may be altered (by a magnitude of order three) due to a 0.3 eV difference in the Gibbs free energy^24,69^. The embedment of extended NGs, e.g., Rh, Tc, V, and Ti, in small N-doped nanographene is expected to improve their HER performance for SACs with hydrogen over-binding (i.e., a larger negative Gibbs free energy value). This is a macrocyclic ligand of the SAC center. In particular, it was observed that the Gibbs free energy of small N-doped nanographene-embedded V was − 0.03. This value is closer to zero compared to other SACs that have been investigated (e.g., Co-NG). It should be mentioned that Tc is a radioactive substance and was incorporated into the study for solely comparison purposes.

In order to realize the origin of the weaker adsorption of H onto small N-doped nanographene-embedded SACs, the d-state centers were examined, finding a downward change as compared to several two-dimensional graphene-embedded SACs.

### Implementation of GWO-ANFIS model

**Figure 5:**
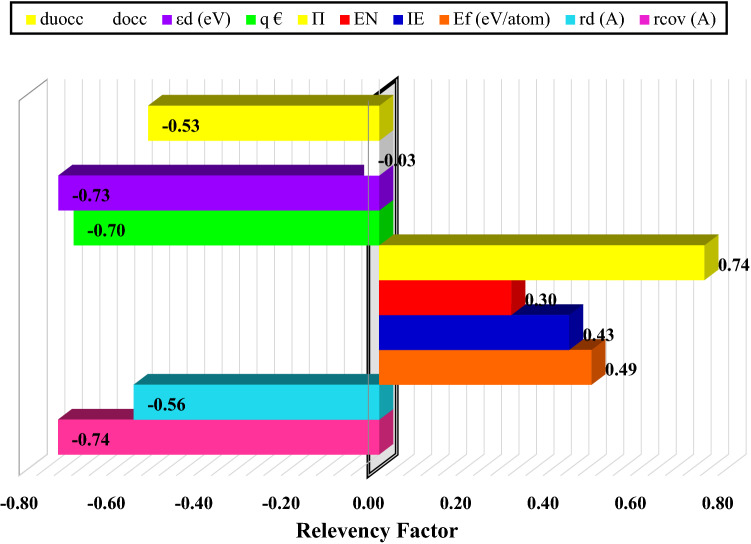
Relevance factor of different input variables.

In this study, the ANFIS approach has been applied to predict hydrogen adsorption energy of different metals in SACs as an essential step in HER. First of all, sensitivity analysis^62^ based on Fig. 5 has been carried out to identify the most important descriptors as models' inputs. These descriptors were the covalent radius [r_cov_ (A)], Zunger radius of the atomic d-orbital [r_d_ (A)], the formation energy of the single-atom site [E_f_ (eV/atom)], ionization energy (IE), electronegativity (EN), the d-band center from − 6 to 6 eV [εd (eV)], number of valence electrons (Π), Bader charge (q €), number of occupied d states from 0 to − 2 eV (d_occ_), and number of unoccupied d states from 0 to 2 eV (du_occ_). As can be seen, the number of valence electrons and the covalent radius are the most effective parameters with a relevancy of 0.74.

**Figure 6:**
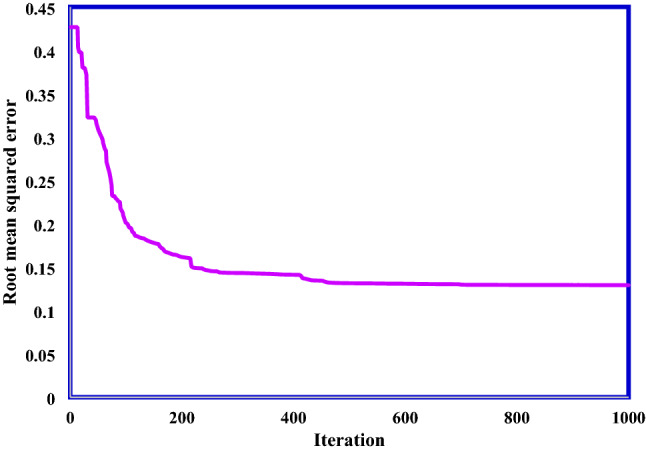
Performance of GWO approach to optimize ANFIS model.

We have used 5 clusters and Gaussian membership functions in the suggested ANFIS model. Accordingly, 110 membership functions parameters should be optimally determined. In this regard, we have used GWO approach to determine optimum membership function parameters. Figure 6 indicated the root mean squared error (RMSE) between the output and experimental values of adsorption energy during 1000 iterations.

**Figure 7:**
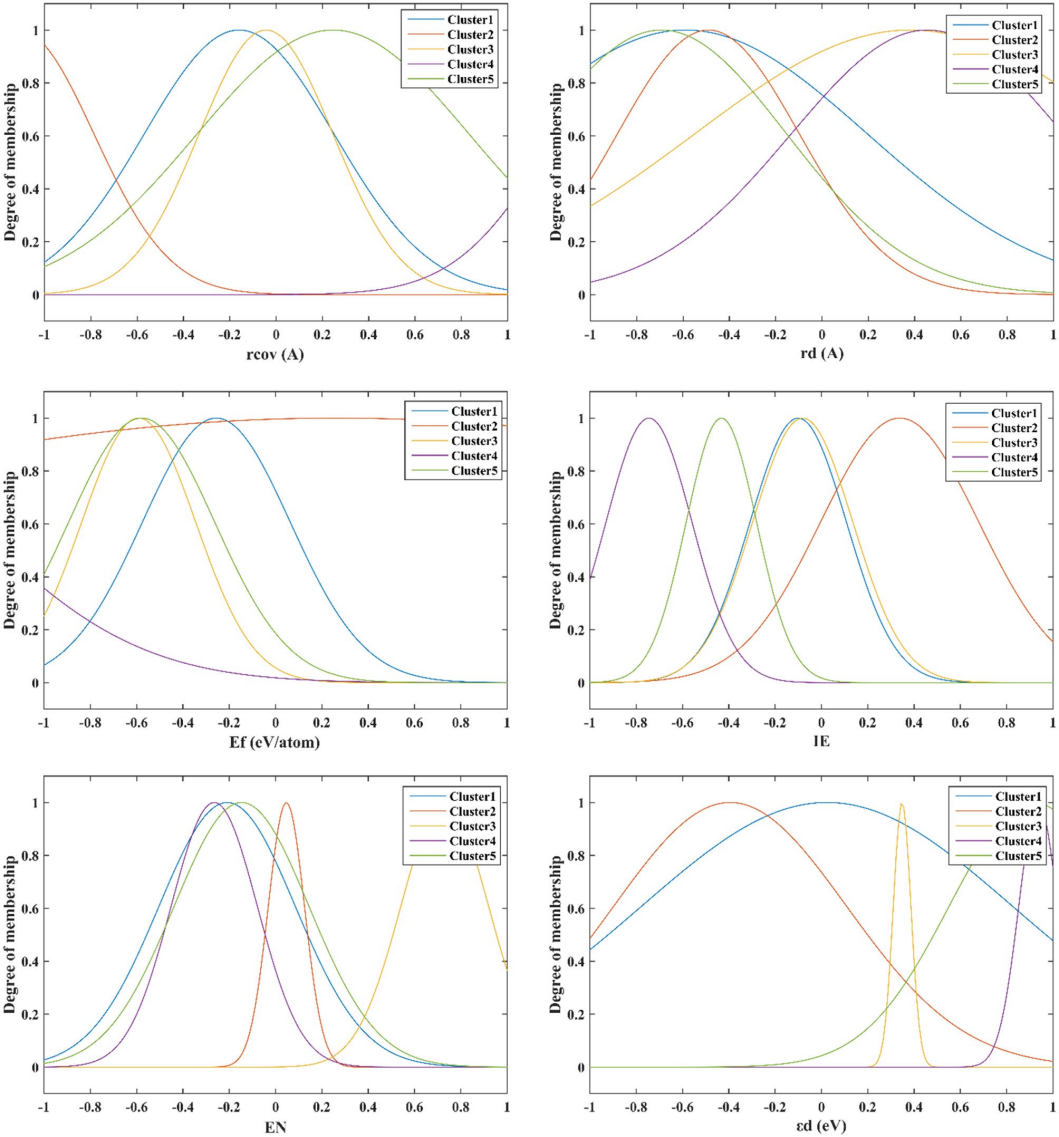

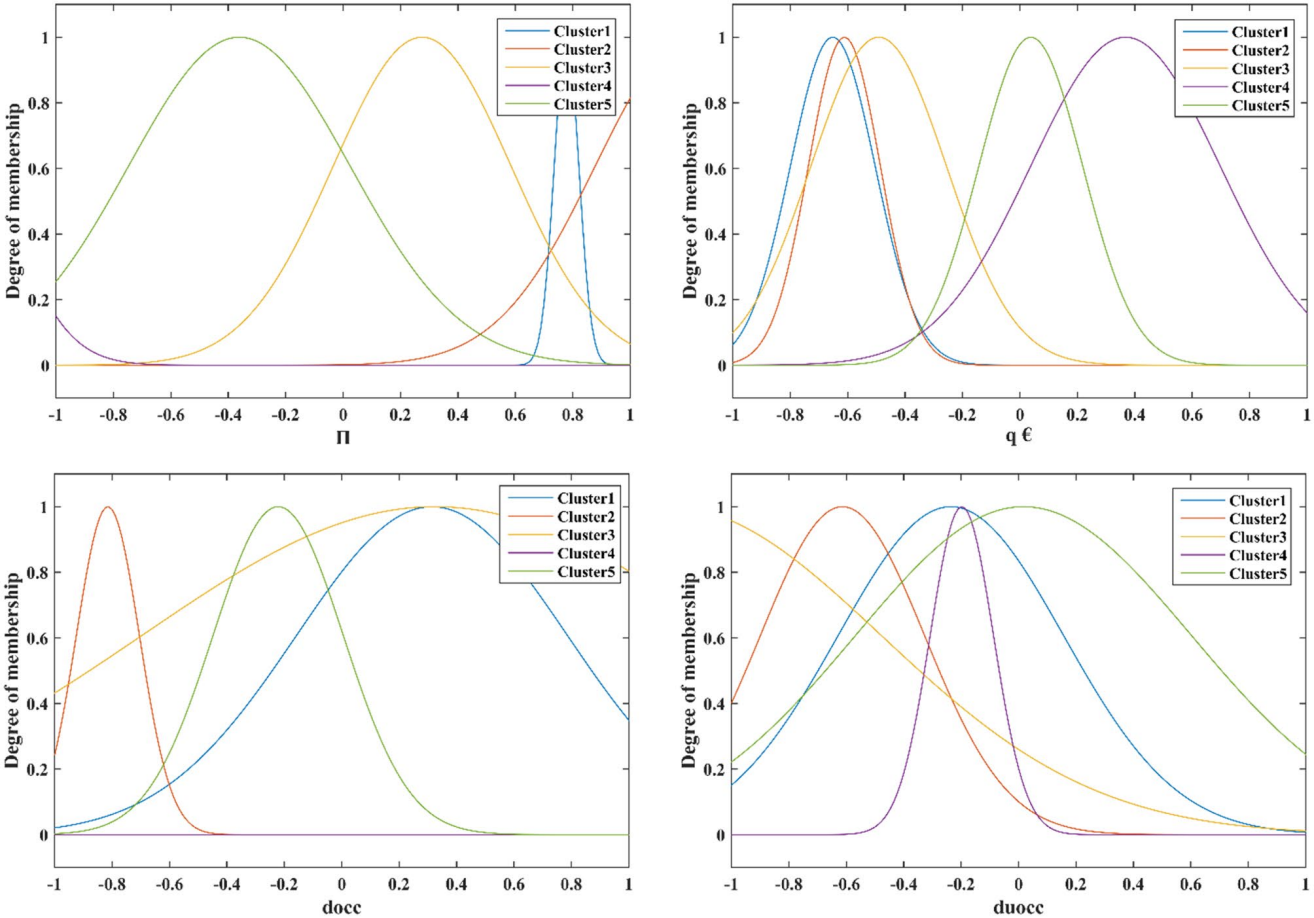
Optimum membership function parameters of the suggested GWO-ANFIS.

In addition, the optimized membership function parameters for all inputs have been indicated in Fig. 7 for different clusters.

Different statistical analyses such as R-squared (R^2^), percentage of mean relative error (MRE%), mean squared error (MSE), root mean squared error (RMSE), and standard deviation (STD) have been reported in Table 1. These values confirm great accuracy of developed GWO-ANFIS model.

**Table 1:** Statistical analyses of GWO-ANFIS model.

Set	R^2^	MRE (%)	MSE	RMSE	STD
Train data	0.989	19.975	0.0124	0.1115	0.0743
Test data	0.967	41.238	0.0293	0.1711	0.1058
Total data	0.984	25.344	0.0167	0.1711	0.0859

**Figure 8:**
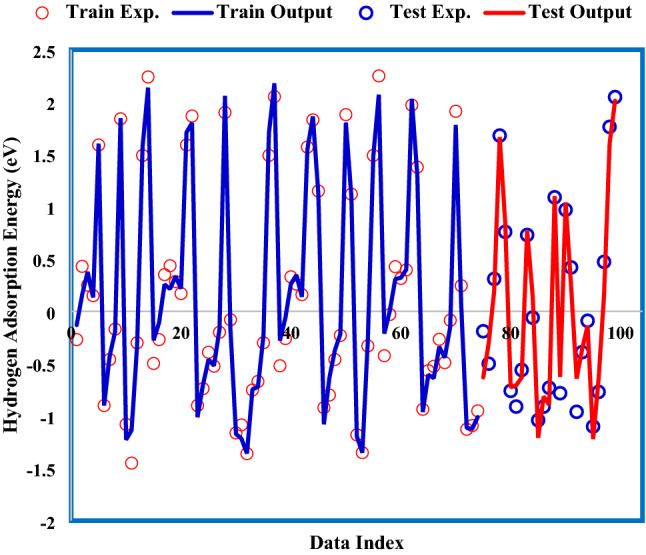
GWO-ANFIS versus experimental hydrogen adsorption energy.

In Fig. 8, the adsorption energies from the GWO-ANFIS model and experiment were displayed simultaneously against the data index. It can be observed that the present model is greatly capable of forecasting the hydrogen adsorption energies.

**Figure 9:**
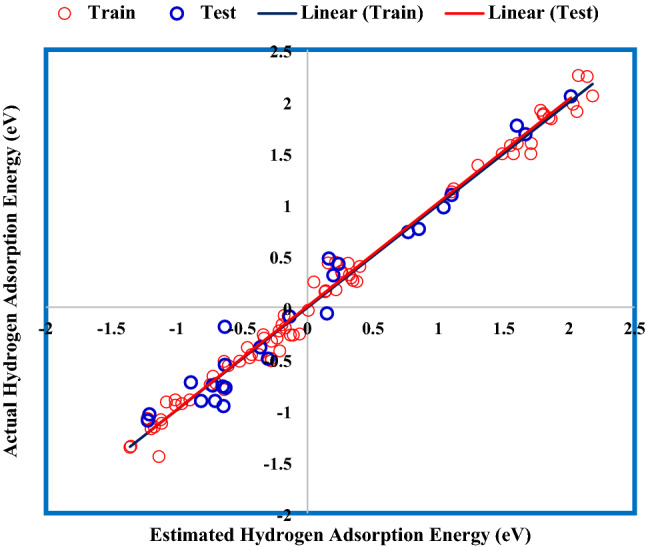
Regression plot between GWO-ANFIS and real data.

Assessing the proximity of GWO-ANFIS values to actual was carried out by investigating the coefficient of determination (R^2^). This parameter varies between 0 and 1, which closeness to unity denotes its high accuracy. Figure 9 displays the cross illustration of actual and GWO-ANFIS outputs. The main portion of adsorption energy values was accumulated around the bisector line, and the obtained R^2^ values for training and testing stages of the GWO-ANFIS were 0.989 and 0.967, respectively; thus, approving the excellent fitness of the GWO-SVM model.

**Figure 10:**
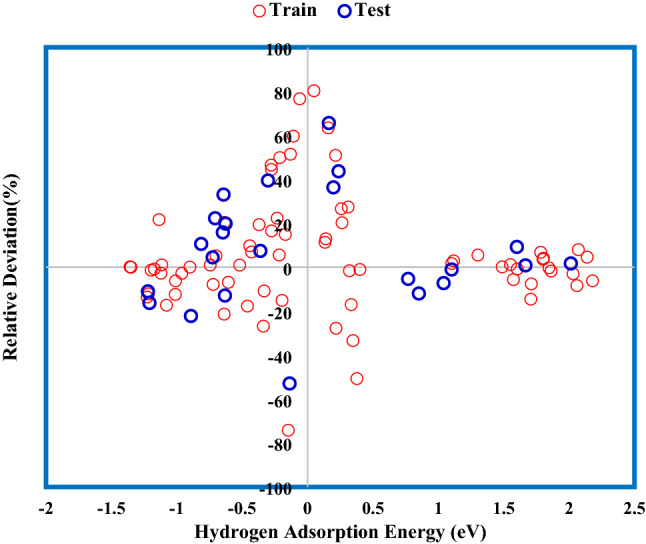
Relative deviation percentage of GWO-ANFIS model.

The relative deviation percentage for the suggested GWO-ANFIS is shown in Fig. 10. The error percentage values were mainly within the 20% band, representing the satisfactory accuracy of the model.

**Figure 11:**
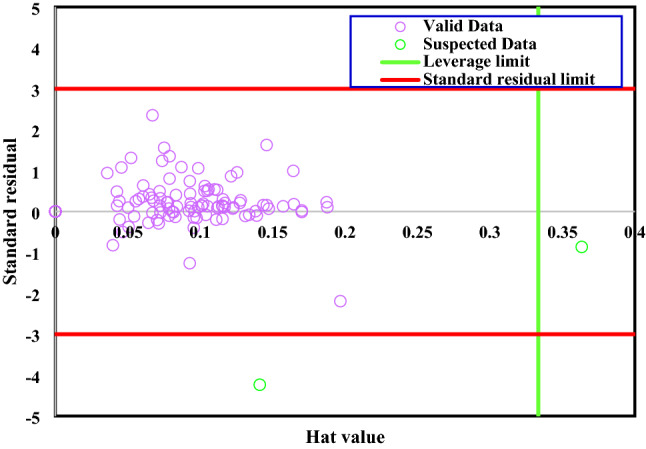
outlier detection analysis for GWO-ANFIS model.

In order to find outliers, the Williams plot has been applied and illustrated in Fig. 11. It can be evidently observed the most of the adsorption energy values except 2 points, located in the range of $\pm 3$ standard residual values, signifying that as well as being satisfactory in statistical analysis, the GWO-ANFIS model could also be used in different conditions.

## Conclusions

In the current study, Adaptive Neuro-Fuzzy Inference optimized by Gray Wolf Optimization (GWO) method was used to predict hydrogen adsorption energy (ΔG ) obtained from density functional theory (DFT) for single transition-metal atoms including Sc, Ti, V, Cr, Mn, Fe, Co, Ni, Cu, Zn, Zr, Nb, Mo, Tc, Ru, Rh, Pd, Ag, Cd, Hf, Ta, W, Re, Os, Ir, Pt, and Au embedded in N-doped carbon of different sizes. The great accuracy of developed GWO-ANFIS with 5 clusters was confirmed by different statistical approaches such as the R-squared and MSE of developed models were 0.967 and 0.029, respectively. In addition, it was found from the sensitivity analysis that the number of valence electrons and the covalent radius is the most effective parameters with the relevancy of 0.74. Consequently, the proposed GWO-ANFIS can be used as a helpful approach to determine hydrogen adsorption energy of different metal/NC structures.
